# Determinants of pH profile and acyl chain selectivity in lysosomal phospholipase A_2_[S]

**DOI:** 10.1194/jlr.M084012

**Published:** 2018-05-03

**Authors:** Vania Hinkovska-Galcheva, Robert Kelly, Kelly A. Manthei, Renee Bouley, Wenmin Yuan, Anna Schwendeman, John J. G. Tesmer, James A. Shayman

**Affiliations:** Department of Internal Medicine, University of Michigan Medical School,* University of Michigan, Ann Arbor, MI; Life Sciences Institute and the Departments of Pharmacology and Biological Chemistry,† University of Michigan, Ann Arbor, MI; Department of Pharmaceutical Science, Biointerfaces Institute, § University of Michigan, Ann Arbor, MI; Department of Biological Sciences,** Purdue University, West Lafayette, IN

**Keywords:** acyltransferase, 1-*O*-acylceramide, lysosome, phospholipase A_2_ group XV, crystallography, cholesterol, ceramide, selectivity, pH dependence

## Abstract

Lysosomal phospholipase A2 (LPLA_2_) is characterized by broad substrate recognition, peak activity at acidic pH, and the transacylation of lipophilic alcohols, especially *N*-acetyl-sphingosine. Prior structural analysis of LPLA_2_ revealed the presence of an atypical acidic residue, Asp13, in the otherwise hydrophobic active site cleft. We hypothesized that Asp13 contributed to the pH profile and/or substrate preference of LPLA_2_ for unsaturated acyl chains. To test this hypothesis, we substituted Asp13 for alanine, cysteine, or phenylalanine; then, we monitored the formation of 1-*O*-acyl-*N*-acetylsphingosine to measure the hydrolysis of *sn*-1 versus *sn*-2 acyl groups on a variety of glycerophospholipids. Substitutions with Asp13 yielded significant enzyme activity at neutral pH (7.4) and perturbed the selectivity for mono- and double-unsaturated acyl chains. However, this position played no apparent role in selecting for either the acyl acceptor or the head group of the glycerophospholipid. Our modeling indicates that Asp13 and its substitutions contribute to the pH activity profile of LPLA_2_ and to acyl chain selectivity by forming part of a hydrophobic track occupied by the scissile acyl chain.

Lysosomal phospholipase A_2_ (LPLA_2,_ PLA2GXV) is characterized by both calcium-independent phospholipase A_2_ and ceramide acyltransferase activities (1–4). It has broad substrate specificity, recognizing several glycerophospholipids, including phosphatidylcholine, phosphatidylethanolamine, phosphatidylglycerol, and phosphatidylserine. In the presence of lipophilic alcohols, such as N-acetyl-sphingosine (NAS), LPLA_2_ acts as an acyltransferase, generating *O*-linked acyl alcohols. If no acceptor other than water is present, LPLA_2_ acts as a phospholipase and only a lysophospholipid and a free fatty acid are formed.

LPLA_2_ is localized to late endosomes and lysosomes and, like other lysosomal hydrolases, has an acidic pH optimum. LPLA_2_ null mice are characterized by the early development of increased surfactant phospholipid levels and alveolar macrophage foam cells, similar to that observed in drug-induced phospholipidosis (5). Other studies have identified a role for LPLA_2_ in host defense (6, 7). The acidic pH is important for the binding of the enzyme to liposomes or membranes and is mediated through a distinct membrane binding domain (8, 9). This binding domain may be the basis by which LPLA_2_ mediates amiodarone-associated phospholipidosis (10). However, the enzyme is active against water-soluble truncated oxidized phospholipids at neutral pH (11), suggesting that some substrates may directly access the catalytic domain, and that there may be a biological role for secreted LPLA_2_ (12).

In an earlier study, the specificities of LPLA_2_ toward *sn*-1 versus *sn*-2 fatty acyl groups were evaluated by separating and identifying distinct species of 1-*O*-acyl-ceramides formed as the product of its lipase and transacylase reactions (13). We reported that both *sn*-1 and *sn*-2 fatty acyl groups of phosphatidylcholine were subject to hydrolysis depending on the fatty acyl species present, with preference for unsaturated acyl chains, which are typically found at the *sn-*2 position of phospholipids. However, at the time of that study, the structure of LPLA_2_ had not yet been discovered and thus, the molecular basis for fatty acid selectivity was unknown.

Recently, our groups reported the structure of LPLA_2_ and LCAT, a structurally related phospholipase A_2_ with transacylase activity (9, 14). The crystal structure confirmed that the catalytic core of LPLA_2_ is an α/β hydrolase domain but with distinct membrane binding and “cap” domains (**Fig. 1A**). The catalytic triad and adjacent oxyanion hole are found at the base of a largely hydrophobic bowl that is at least partially shielded from solvent by a loop that extends from the cap domain, and is the binding site for glycerophospholipids and lipophilic alcohols (Fig. 1B). Both the lipase and acyltransferase reactions of LPLA_2_ proceed sequentially with the transient acylation of the catalytic serine by the scissile fatty acyl group. The same lipid binding pocket is anticipated to be used for the departing lysophospholipid product and the ultimate lipophilic alcohol that serves as an acyl group acceptor.

**Fig. 1.**
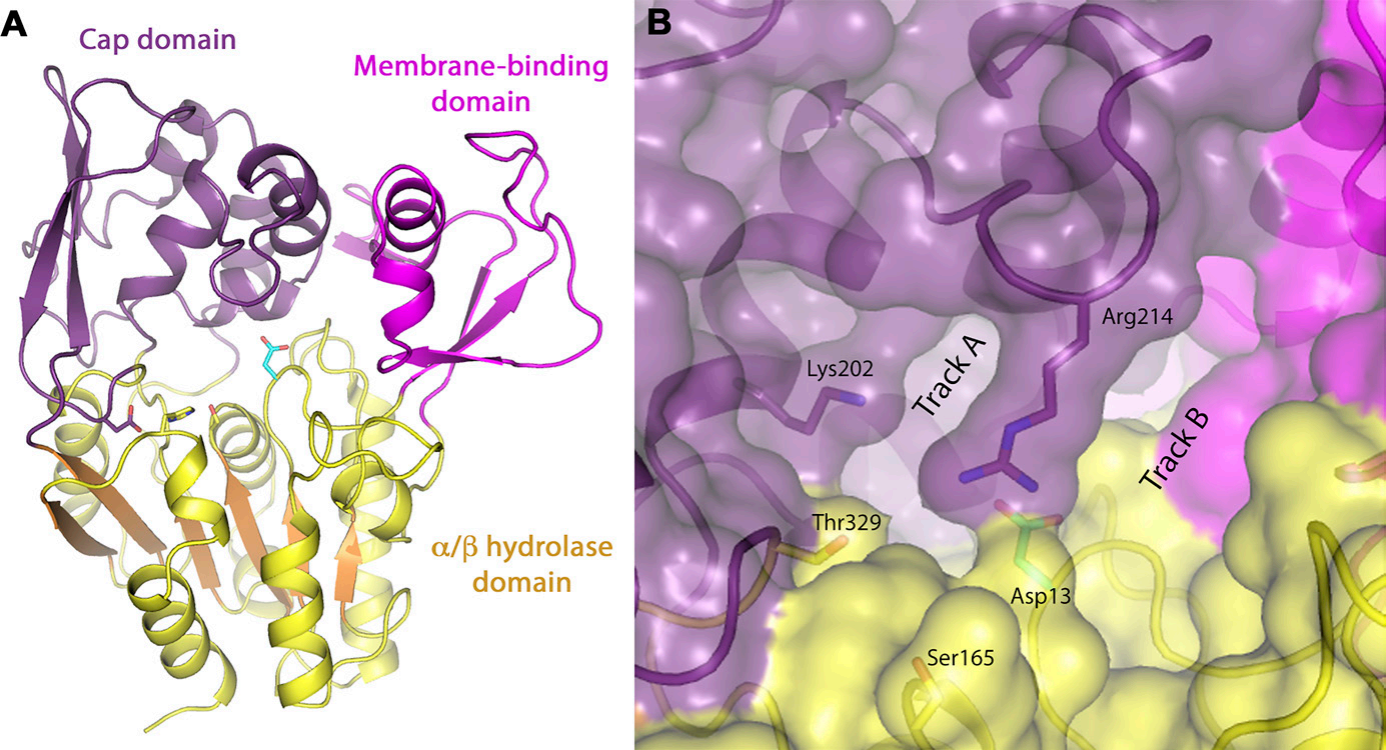
LPLA_2_ tertiary structure. A: The enzyme is composed of catalytic α/β hydrolase (show in yellow with orange β-sheets), membrane binding (magenta), and cap (purple) domains. The side chains of the catalytic triad composed of Ser165, Asp 327, and His359 are shown. The side chain of Asp13 is shown in cyan. B: The proposed locations of tracks A and B are shown in relation to the catalytic serine Ser165 and Asp13, a position typically conserved as a large hydrophobic residue in other related lipases.

Based on these structures, substrate modeling, and the position of disease-causing mutations in LCAT (9, 15), we proposed that the orientation of the bound phospholipid in the active site underlies the specificity of LPLA_2_ for fatty acids in the *sn*-2 vs. the *sn*-1 position. We further proposed that the observed acyl chain length preference of LPLA_2_ is determined by two hydrophobic grooves, termed the A and B tracks, leading away from the catalytic triad of the enzyme, with the A track likely to be that occupied by the scissile acyl chain.

Asp13 is a conspicuous residue located next to the oxyanion hole and contributes to the A track in a position typically conserved as hydrophobic residue (M/V/L) in type I lipases (16–18). The analogous residue in LCAT is Cys31, which is also atypical. We hypothesized that the pH 4.5 optimum of LPLA_2_ in part reflects the requirement for protonation of this side chain at low pH, resulting in its neutralization, and allowing acyl chains to bind in this track. We further hypothesized that Asp13 contributes to substrate preference for unsaturated acyl chains.

These hypotheses were tested by substituting Asp13 for Ala (D13A), Cys (D13C), or Phe (D13F) and by the measurement of transacylase and lipase activities as a function of pH. We observed that the D13F variant exhibits significantly less pH dependence with an observed augmention of phosholipase A activity at pH 7.4. We also observed a role for Asp13 in acceptor specificity with regard to unsaturated fatty acyl groups independent of their *sn*-1 vs. *sn*-2 positions. These studies further confirmed that the scissile fatty acyl group resides in track A and confers either phospholipase A_1_ or A_2_ activity based on the affinity of the fatty acyl group for this track.

## METHODS

### Reagents

1,2-Dipalmitoyl-*sn*-glycero-3-phosphocholine (DPPC), 1,2-distearoyl-*sn*-glycero-3-phosphocholine (DSPC), 1,2-dioleoyl-*sn*-glycero-3-phosphocholine (DOPC), 1-oleoyl-2-myristoyl-*sn*-glycero-3-phosphocholine (OMPC), 1-palmitoyl-2-oleoyl-*sn*-glycero-3-phosphocholine (POPC), 1-palmitoyl-2-linoleoyl-*sn*-glycero-3-phosphocholine (PLPC), 1-palmitoyl-2-arachidonoyl-*sn*-glycero-3-phosphocholine (PAPC), 1-palmitoyl-2-docosahexanoyl-*sn*-glycero-3-phosphocholine (PDPC), 1-oleoyl-2-palmitoyl-*sn*-glycero-3-phosphocholine (OPPC), 1-oleoyl-2-steraroyl-*sn*-glycero-3-phosphocholine (OSPC), 1-palmitoyl-2-oleoyl-*sn*-glycero-3-phosphoethanolamine (POPE), 1-palmitoyl-2-oleoyl-*sn*-glycero-3-phospho-(1′rac-rlycero) Na salt (POPG), 1-palmitoyl-2-oleoyl-*sn*-glycero-3-phospho-L-serine (POPS), 1-palmitoyl-2-oleoyl-*sn*-glycero-3-phosphate (POPA), 1-stearoyl-2-oleoyl-*sn*-glycero-3-phosphocholine (SOPC), 1- oleoyl -2-stearoyl-*sn*-glycero-3-phosphocholine (OSPC), 1-stearoyl-2-linoleoyl-*sn*-glycero-3-phosphocholine (SLPC), 1-stearoyl-2-linoleoyl-*sn*-glycero-3-phosphate (SLPA), 1-stearoyl-2-oleoyl-*sn*-glycero-3-phosphoethanolamine (SOPE), 1-stearoyl-2-oleoyl-*sn*-glycero-3-phospho-L-serine (SOPS), 1-stearoyl-2-oleoyl-*sn*-glycero-3-phosphate (SOPA), 1-stearoyl-2-oleoyl-*sn*-glycero-3-phospho-(1′rac-glycerol) (SOPG), 1-stearoyl-2-linoleoyl-*sn*-glycero-3-phosphate (SLPA), 1-stearoyl-2-linoleoyl-*sn*-glycero-3-phospho-(1′rac-glycerol) (SLPG), 1-stearoyl-2-linoleoyl-*sn*-glycero-3-phospho-L-serine (SLPS), 1-stearoyl-2-linoleoyl-*sn*-glycero-3-phosphoethanolamine (SLPE), 1-palmitoyl-2-stearoyl-*sn*-glycero-3-phosphocholine (PSPC), *N*-acetyl-D-*erythro*-sphingosine (NAS), 1,2-di-*O*-octadecenyl-*sn*-glycero-3-phosphocholine (DODPC), 1-O-hexadecyl-2-acetyl-sn-glycerol (HAG) were purchased from Avanti (Alabaster, AL). Purified recombinant mouse LPLA2 was obtained from Proteos Inc. (Kalamazoo, MI). Monoclonal antibodies against LPLA_2_ were developed from cloned recombinant protein, and purified from Maine Biotechnology Services (Portland, ME). Anti-His-antibody, BCA protein assay reagent, 1-*O*-hexadecyl-rac-glycerol (HG), oleoylethanolamide (OEA), and anandamide (AEA) were from Sigma (Rockford, IL). Goat anti-rat IgG-HRP was from Santa Cruz Biotechnology (Dallas, TX). Dicetyl phosphate and polyethylenimine (average MW 25 kDa, and degree of polymerization 580) were from Sigma (St. Louis, MO). HPTLC silica gel plates (10 × 20 cm) were from Merck KGaA (Darmstadt, Germany).

### Expression and purification of LPLA_2_

LPLA_2_ variants were expressed and purified as previously described (9). The pcDNA4 plasmid containing the human LPLA_2_ gene with codons optimized for expression in HEK293F cells was employed. The construct encodes the LPLA_2_ signal sequence, followed by a 6xHis tag and tobacco etch virus protease cleavage site, and then the sequence corresponding to mature LPLA_2_ (pcDNA4-LPLA_2_). For expression of LPLA_2_, HEK293F cells were grown in suspension in Gibco FreeStyle media supplemented with 0.5% fetal bovine serum (Fisher Scientific, Pittsburgh, PA). Upon attaining a cell density of 1.5×10^6^/ml, the cells were transiently transfected using a pcDNA4-LPLA_2_:polyethylenimine molar ratio of 1:2. Conditioned media was harvested 5 d later, supplemented with HEPES pH 7.5 to a final concentration of 50 mM, and then loaded on a 3 ml Ni-NTA column. The column was washed with 10 ml buffer containing 20 mM HEPES pH 7.4, 300 mM NaCl, and 10 mM imidazole pH 8, and eluted using the same buffer containing 200 mM imidazole pH 8. The eluate was dialyzed overnight at 4°C against 20 mM HEPES pH 7.5, 100 mM NaCl, and 1 mM DTT using a dialysis cassette G2 20,000 MWCO (Thermos Scientific Rockford, IL). The protein was concentrated to 0.5–1 mg/ml using Amicon Ultra 4 centrifuge filters (Merck KGaA, Darmstadt Germany). Protein concentrations were determined by use of the Bradford reagent and confirmed by a NanoDrop spectrophotometer read at A280. Protein purity was monitored using SDS-PAGE and Western analysis with anti-LPLA_2_ and anti-His antibodies.

### Site-directed mutagenesis

Site-directed variants of pcDNA4-LPLA_2_ were made using a single primer for each substitution and Q5 Polymerase (New England Biolabs, Ipswich, MA): D13A,GGTCCTGGTGCCCGGCGCCCTGGGGAATCAGCTGG; D13C, GGTCCTGGTGCCCGGCTGTCTGGGGAATCAGCTGG; D13F, CCTGGTGCCCGGCTT­CCTGGGGAATCAGC; L49F, GGCTGAACCTGGAACTGTTCCTGCCAGTCATCATTGAC; V101L, GACCCTTCCAAAAGCTCCGTGGGATCTTACTTCCACACTATG; N213Q/R214G, CTGGCTAGTGGCGATAACCAGGGCATCCCAGTCATTGGGCC; I360L, CCAGGAAGCGAACATCTGGAAATGCTGGCTAACGC.

In selected experiments, multiple substitutions were made. The “total LCAT D13C variant” (ToLCC) denotes D13C, L49F, V101L, N213Q, R214G, and I360L substitutions. The “total LCAT D13F variant” (ToLCF) denotes D13F, L49F, V101L, N213Q, R214G, and I360L substitutions.

### Liposome pull-down assay

Liposomes consisting of DOPC or DODPC and sulfatide (10:1, molar ratio, 127 µM total) were incubated with 5 µg of LPLA_2_ variants in 500 µl of 48 mM Na citrate pH 4.5, or 50 mM HEPES buffer pH 7.4 for 30 min on ice. The reaction mixture was then centrifuged for 1 h at 150,000 *g* at 4°C. The resulting precipitate was rinsed with cold 50 mM Na citrate pH 4.5/50 mM HEPES buffer (pH 7.4) and dissolved with 40 µl of SDS-PAGE sample buffer. The sample was separated by using 10% SDS-PAGE. After electrophoresis, LPLA_2_ was detected with Coomassie Brilliant Blue. Band quantification was performed with the ImageJ software I1.651j8 (9) (National Institutes of Health).

### LPLA_2_ esterase assay

*p*-Nitro-phenylbutyrate (pNPB) was used to measure directly the catalytic activity of LPLA_2_ on a soluble ester substrate (8). pNPB (Sigma, St. Louis, MO) was diluted to 10 mM using the reaction buffer (20 mM HEPES pH 7.5, 150 mM NaCl) containing 10% DMSO, and the reaction was initiated by the addition of 60 µl of 0.1 µM LPLA_2_ to 10 µl of pNPB. Release of *p*-nitrophenoxide was monitored by increased absorbance at 400 nm on a Spectramax plate reader.

### Transacylase activity of LPLA_2._

The transacylase reaction is based on the ability of LPLA_2_ to transfer an acyl group from the *sn*-2 or *sn*-1 position of a glycerophospholipid to NAS. Formation of 1-*O*-acyl-NAS is unique to the LPLA_2_ reaction (19). The reaction mixture contained liposomes (127 μM of phospholipid), buffer (48 mM Na citrate buffer pH 4.5 or 50 mM HEPES pH 7.4), 10 μg/ml BSA, and LPLA_2_ protein (30 ng or 60 ng) in a total volume of 0.5 ml. Liposomes consisting of phospholipid substrate/sulfatide/NAS (10:1:3 molar ratio) were used. LPLA_2_ binds preferentially to negatively charged liposomes. Sulfatide was used in the liposomes as it is not itself a substrate and does not function as a cofactor for lysosomal hydrolases. The reaction was initiated by addition of LPLA_2_, carried out at 37°C for different periods of time (as shown in the figure legends), and terminated by the addition of 3 ml chloroform/methanol (2/1, v/v), followed by 0.3 ml of 9% (w/v) NaCl. After centrifugation for 7 min at 1800 *g*, the resulting lower layer was transferred to a new tube and dried under a stream of nitrogen gas. The dried lipid was dissolved in 40 μl chloroform/methanol (2/1, v/v) and applied to HPTLC plates. HPTLC plates were run in chloroform/acetic acid (9/1, v/v), chloroform/methanol/acetic acid (90/1/5, v/v/v), or chloroform/methanol/acetic acid (95/2/5, v/v/v) as designated. The plates were dried and soaked in 8% (w/v) CuSO_4_.5H_2_O, 6.8% (v/v) H_3_PO_4_, and 32% (v/v) methanol, and then charred for 15 min in a 150°C oven. For the argentation of HPTLC plates, 10% AgNO_3_ in acetonitrile was used. Plates were immersed for 10 min, dried, and then activated for 30 min at 100°C. To prevent the silica gel from peeling off during subsequent steps, the dried plate was incubated in 20% (v/v) methanol containing 0.5% (v/v) acetic acid to remove AgNO_3_. The plate was then soaked in CuSO_4_ solution, dried, and charred as described above. Scanned plates were analyzed by ImageJ 1.651j8 (National Institutes of Health).

### Measurement of lipase activity under acidic and neutral conditions

The assay was performed as previously described (11). The reaction mixture included liposomes consisting of DODPC and DOPC (molar ratio 2.4:1). Buffer containing 50 mM Na citrate pH 4.5 or 50 mM HEPES pH 7.4 were used. The reaction was initiated by the addition of LPLA_2_ variants (30 ng) in a final volume of 500 µl. The reaction proceeded at 37°C and was terminated by the addition of 3 ml of chloroform/methanol (2:1) and 0.3 ml of 0.9% (w/v) NaCl. The mixture was centrifuged at 800 *g* for 5 min at room temperature. The resultant lower organic layer was transferred into another glass tube and dried down under a stream of nitrogen gas. The dried lipid was dissolved in 40 µl chloroform/methanol (2:1), applied to an HPTLC plate, and developed in a solvent consisting of chloroform/methanol/pyridine (98/2/0.5, v/v/v). The plate was dried and soaked in 8% (w/v) CuSO_4_, 5H_2_O, 6.8% (v/v) H_3_PO_4_, and 32% (v/v) methanol. The uniformly wet plate was briefly dried with a hair dryer and then charred for 15 min in a 150°C oven. The plate was scanned and the content of the product fatty acid was estimated using ImageJ 1.651j8v software (National Institutes of Health).

### LCAT activity assay

The sterol esterification activity of recombinant human LCAT (rhLCAT) or LPLA_2_ variants was measured using dehydroergosterol (DHE), a naturally occurring fluorescent sterol, as the substrate in combination with cholesterol oxidase (20). Peptide-DHE-synthetic high density lipoprotein (sHDL) samples were prepared via the thin-film method. Briefly, DPPC, POPC, and DHE (molar ratio 9:9:2) were dissolved in chloroform. The ApoA1 22-mer peptide 22A, ESP24218, (sequence PVLDLFRELLNELLEALKQKLK) was dissolved in 1:1 (v/v) methanol/water, and then mixed with the lipid/chloroform solution (21). The solvent was removed under a flow of nitrogen for 2 h and then overnight in a vacuum oven. The lipid film was rehydrated with 20 mM phosphate buffer containing 1 mM EDTA (pH 7.4), followed by water bath sonication for 10 min, and probe sonication (2 min × 50 W) to obtain clear DHE-sHDL. All steps were performed at room temperature. The final DHE concentration in peptide-DHE-sHDL was 0.5 mM. The size of the peptide-DHE-sHDL was detected via dynamic light scattering. Briefly, the sHDL samples were diluted by PBS (pH 7.4) to a final peptide concentration of 1 mg/ml and then measured by Zetasizer Nano ZSP (Malvern Instruments, Malvern, UK). The volume size for the peptide-DHE-sHDL substrates was 9.5 ± 0.08 nm.

The DHE assay was performed in 96-well white polystyrene plates in triplicate. Briefly, 100 μl of 0, 5, 10, 20, 40, 60, and 100 μM DHE-containing sHDL substrates in assay buffer (PBS pH 7.4 plus 1 mM EDTA, 5 mM β-mercaptoethanol, and 60 µM albumin) preheated to 37°C were incubated with 100 μl of 5 μg/ml rhLCAT or LPLA_2_ protein in assay buffer lacking β-mercaptoethanol preheated to 37°C. The plates were incubated at 37°C with shaking (80 rpm/min) for 0, 10, and 20 min. The reactions were stopped by addition of 50 μl of 3.75 U/ml cholesterol oxidase in PBS containing 1 mM EDTA and 7% Triton X-100. The plates were incubated at 37°C with shaking (80 rpm/min) for another 1 h to quench the fluorescence of unesterified DHE. The fluorescence was measured at an excitation wavelength of 325 nm and an emission wavelength of 425 nm using a SynergyTM NEO HTS Multi-Mode Microplate Reader. The initial velocity (V_o_, μM DHE-ester/h) was taken to be the linear range of DHE-ester concentration versus time. The V_max_ and K_m_ were obtained by plotting V_o_ versus DHE concentration in GraphPad Prism 7 (nonlinear regression, Michaelis-Menten model).

### Docking of ligand substrates into the active site of LPLA_2_

Docking was performed using chain A of the previously published apo-LPLA_2_ crystal structure (PDB entry 4X90), and a model of LPLA_2_ D13F generated based on the conformation of Tyr31 in the crystal structure of the C31Y mutant of LCAT (PDB entry 4XWG) (9, 22). eLBOW (23) was used to perform geometry optimization of each ligand, and subsequently docking was performed with AutoDock Vina (24).

### Protein thermal stability studies

The thermal stability assay was employed to determine the melting point (T_m_) of the expressed proteins as described (25). An incubation mixture consisting of 2.5 μl of 8x SYPRO Orange, 1 μg of LPLA_2_ variants, either 48 mM Na citrate pH 4.5 or 50 mM HEPES pH 7.4, and ddH_2_O in a final volume of 20 μl was added to wells of a 48-well thin-wall PCR plate. The plates were sealed with Optical-Quality Sealing Tape (Bio-Rad) and heated in a Real-Time PCR Detection System Life Technology from 20 to 90°C in steps of 0.2°C. T_m_ values were calculated as the inflection point of the melting curve using instrument software.

### Statistical analysis

Data from at least three independent experiments were analyzed with a paired *t*-test in GraphPad Prism 7 and expressed as mean ± SEM. The differences between control and treated samples were considered statistically significant at *P* < 0.05.

## RESULTS

### LPLA_2_ variant characterization

The D13A, D13C, and D13F variants of LPLA_2_ were created to test the contribution of Asp13 to the pH profile and acyl chain selectivity of the enzyme. D13F restores the position to what it is commonly found in distantly related bacterial lipases. The ToLCC variant incorporates substitutions that alter side chains in the active site to those found in LCAT, which has a pH optimum of 8 (26), including a D13C substitution. ToLCF is the same set of mutations as in ToLCC except with D13F. WT and variant LPLA_2_ were secreted from mammalian cells and purified to homogeneity as confirmed by SDS-PAGE and Western blotting, using both anti-LPLA_2_ and anti-His antibodies (data not shown).

To assess the structural integrity of the LPLA_2_ variants, the thermal stability of each variant was measured at both pH 4.5 and 7.4. At pH 4.5, the D13A, D13C, and D13F variants exhibited <2° decreased T_m_ relative to WT (T_m_ of 65.6 ± 1.4°C), indicating that global folding was not disrupted (**Table 1**). The ToLCC (T_m_ of 61.3 ± 2°C) and ToLCF (T_m_ of 57.3 ± 1.3°C) were, however, significantly less stable, exhibiting ΔT_m_ values of 4.3 and 8.3°C, respectively, less than WT, suggesting significant structural perturbation.

**TABLE 1.** Thermal stability and membrane binding of LPLA_2_ variants at pH 4.5 and 7.4

LPLA_2_ variant	T_m_°C	ΔT_m_	Binding at pH 4.5 (% WT)	Binding at pH 7.4 (%WT at pH 4.5)
WT	65.6 ± 1.4	—	100 ± 6.5	9.5 ± 1.3
D13A	64.5 ± 1.0	−1.1	86 ± 8.7*	16.0 ± 3.0*
D13C	64.9 ± 2.5	−0.7	69 ± 8.3*	9.0 ± 1.8
D13F	63.3 ± 1.0	−2.3	93 ± 11	20.0 ± 1.6**
To LCC	61.3 ± 2.0	−4.3	69 ± 5.0*	9.0 ± 2.6
To LCF	57.3 ± 1.3	−8.3	68 ± 22*	10.0 ± 3.4

T_m_ is defined as the inflection point of the melting curve and was calculated by the Boltzmann equation (32). Membrane binding was measured by the liposome pull-down assay as described in the Methods. The data represent the mean ± SD (n = 3) per measurement, *P < 0.05, **P < 0.001 using a *t*-test.

To assess whether the substitutions affected binding to membranes, the cosedimentation of each LPLA_2_ variant with liposomes was measured (Table 1). At pH 4.5, all variants except D13F demonstrated significantly less cosedimentation than WT (up to 32%). At pH 7.4, the cosedimentation of D13F with liposomes was the only variant that differed from WT (2-fold higher), but still only to a level of roughly 20% of binding of WT at pH 4.5.

**Fig. 2.**
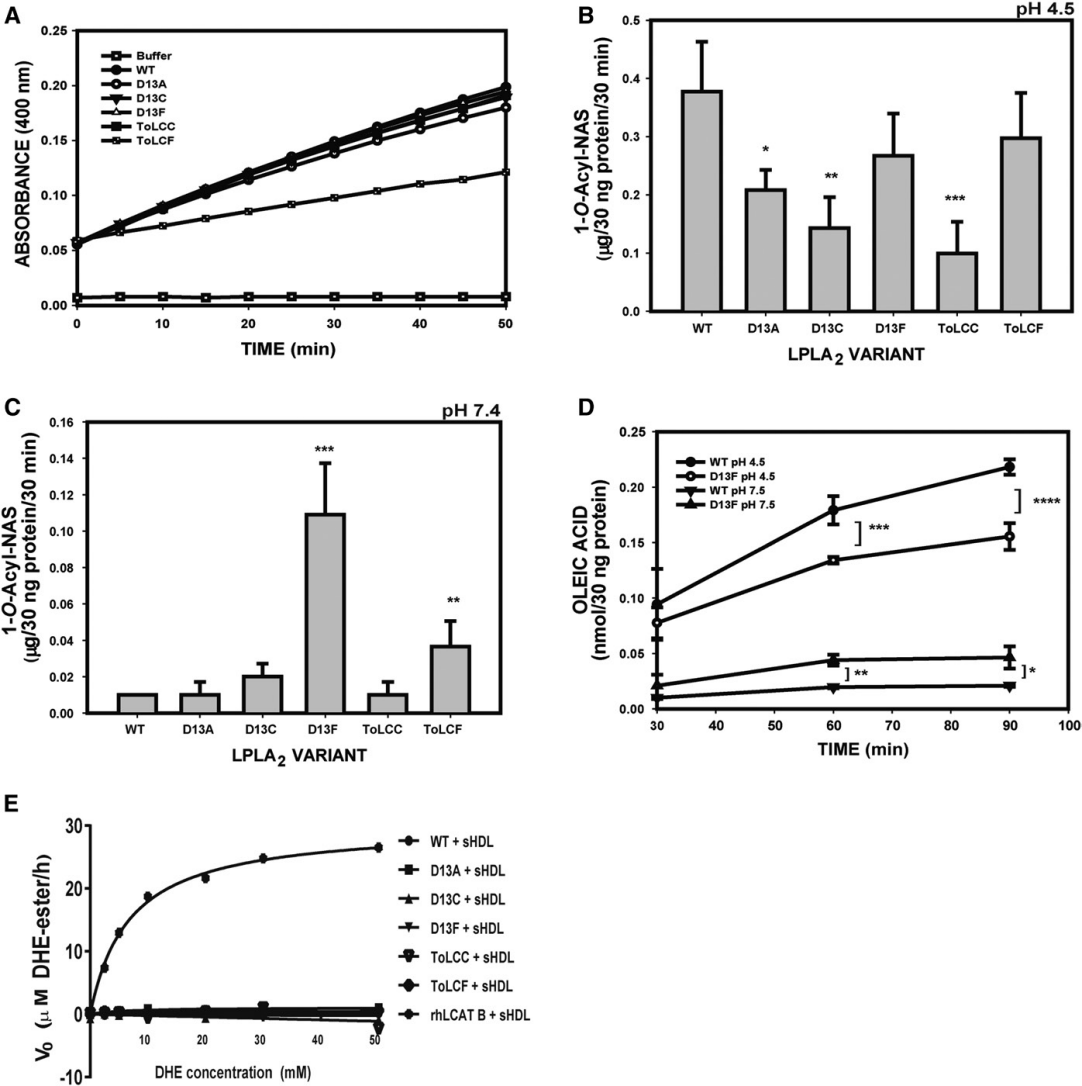
Functional characterization of LPLA_2_ variants. A: Esterase activity measured with pNPB at pH 7.4 of WT and LPLA_2_ variants. Transacylase activities of LPLA2 were determined using DOPC/sulfatide/NAS liposomes (10:1:3 molar ratio) in 48 mM Na citrate pH 4.5 (B) or in 50 mM HEPES pH 7.4 (C), and incubated with 30 ng protein of each variant for 30 min at 37°C. The HPTLC plates were developed in a solvent system consisting of chloroform /acetic acid 9/1 v/v). Ceramide standards were used to calculate activity. D: Lipase activity of LPLA2. The reaction mixture employed liposomes consisting of DODPC and DOPC (molar ratio, 2.4:1) in 50 mM Na citrate pH 4.5 or 50 mM HEPES pH 7.4. The reaction was initiated by adding 30 ng protein of LPLA2 variant to a final volume of 500 μl for different times at 37°C. The mean activities were expressed as nmol oleic acid/30 ng of protein. E: LCAT activity of rhLCAT and LPLA2 variants. Sterol esterification activity was measured using DHE in combination with cholesterol oxidase. The values for all graphs represent the mean ± SD (n = 3) per time point, *P < 0.05, **P < 0.01, ***P < 0.001.

The esterase activity of LPLA_2_ against pNPB has been shown to be independent of both pH and liposome binding (8) and thus, is a good measure of catalytic competency for each variant. With the exception of the ToLCF variant, all variants exhibited hydrolytic activities that were no different from WT (**Fig. 2A**). The loss of esterase activity in ToLCF, taken along with the fact that its T_m_ was the lowest of all the variants, is consistent with substantial disruption of the active site cavity.

Given that all the variants, with the possible exception of ToLCF, seemed to be properly folded and retained substantial activity against at least soluble substrates, we compared their acyltransferase activities at acidic and neutral pH using DOPC as the phospholipid substrate and NAS as the acceptor. Whereas the D13A, D13C, and ToLC variants exhibited decreased 1-*O*-acyl-NAS formation at pH 4.5, the transacylase activities of the D13F and ToLCF variants were not significantly different from WT (Fig. 2B). The transacylase activity of WT was very low at pH 7.4, as previously observed (27), but this activity increased more than 8-fold in the D13F and 4-fold in the ToLCF variant (Fig. 2C). D13C exhibited slightly increased activity compared with WT LPLA_2_ at pH 7.4. Because the D13F and ToLCF substitutions did not result in enhanced activity at pH 4.5, these results are consistent with Asp13 protonation being an important contributor to the overall pH dependence of WT LPLA_2_. Interestingly, ToLCF was not defective in the transacylase assay as it was in the esterase assay, which may indicate that lipophilic substrates and/or environments can stabilize this variant. The lipase activity of WT LPLA_2_ and D13F were also measured as the amount of oleic acid released with the exclusion of N-acetylsphingosine as the fatty acyl acceptor. As in the transacylase assay, the lipase activity of the D13F variant was significantly decreased at pH 4.5, but increased at pH 7.4 compared with WT enzyme (Fig. 2D).

The ToLCC and ToLCF variants represent the introduction of side chains found in the LCAT active site into LPLA_2_. To ascertain whether these substitutions were sufficient to change the acceptor specificity of LPLA_2_, the acyltransferase activity using recombinant HDL particles formed with the ApoA ESP242181 22-mer peptide to a cholesterol-like acceptor was assayed as the formation of acyl-DHE (Fig. 2E). No activity was observed for WT LPLA_2_ or for any of the LPLA_2_ variants, indicating that LCAT selectivity for the cholesterol acceptor at least additionally involves residues outside the active site.

**Fig. 3.**
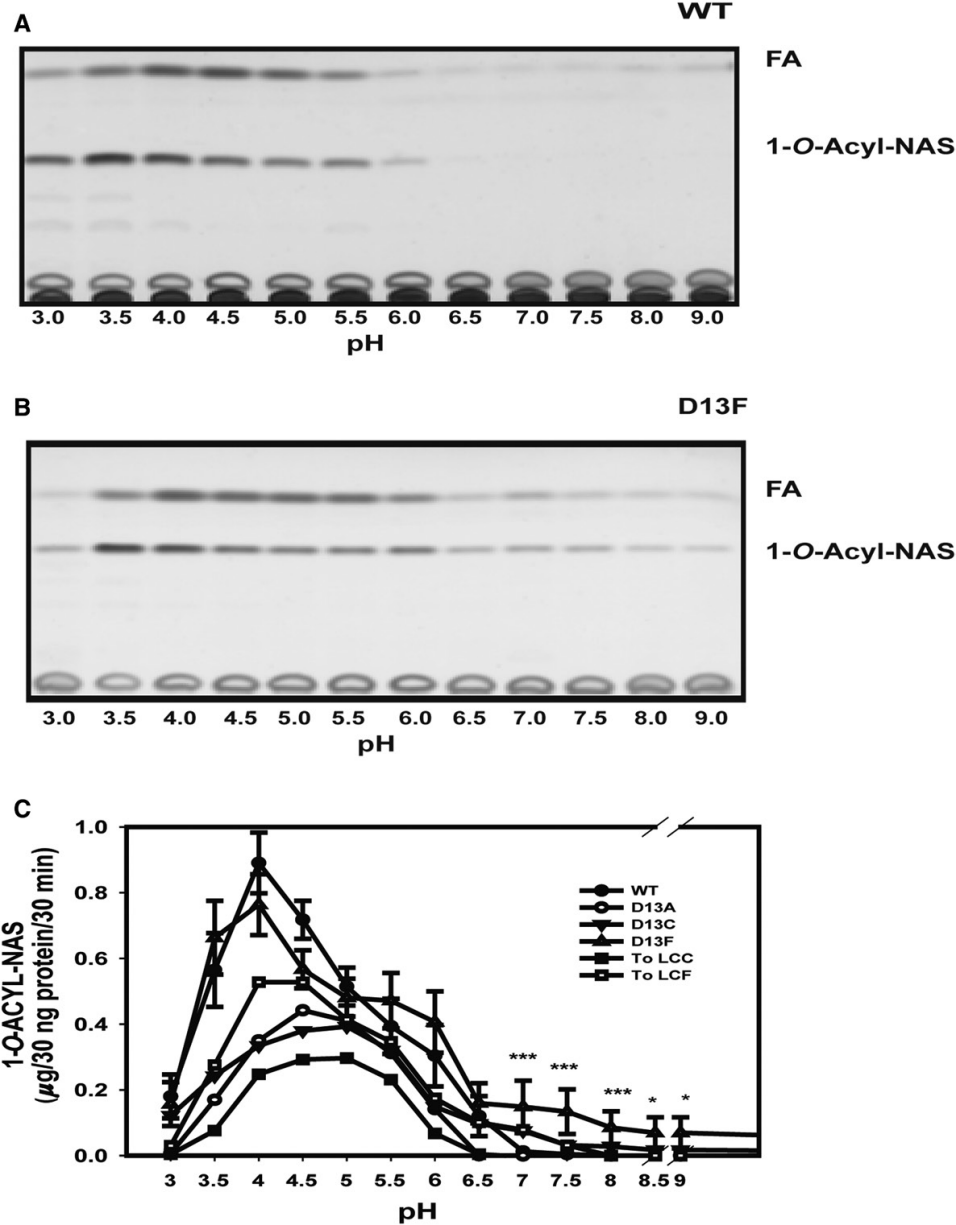
pH dependence of transacylase activity. A: WT LPLA_2_ reaction products separated by TLC, including free fatty acid (FA) and 1-*O*-acyl-NAS as a function of pH. B: D13F reaction products separated by TLC of as a function of pH. C: pH dependent ceramide transacylase activities of individual LPLA_2_ variants. The data represent the mean ± SD (n = 3) per time point, *P < 0.05, ***P < 0.001 using a *t*-test. In all panels, the transacylase activity was determined by incubating DOPC/sulfatide/NAS liposomes (10/1/3 molar ratio) in 48 mM Na citrate pH 4.5 or 50 mM HEPES pH 7.4, with 30 ng of each variant for 30 min at 37°C.

Finally, the pH dependence of the transacylase activity of each LPLA_2_ variant was assayed over a broad pH range using either 50 mM Na citrate or 50 mM HEPES buffer. The D13F, D13C, and ToLCF variants were the only ones that retained activity above pH 6 (**Fig. 3**).

### Positional selectivity of Asp13 LPLA_2_ variants

**Fig. 4.**
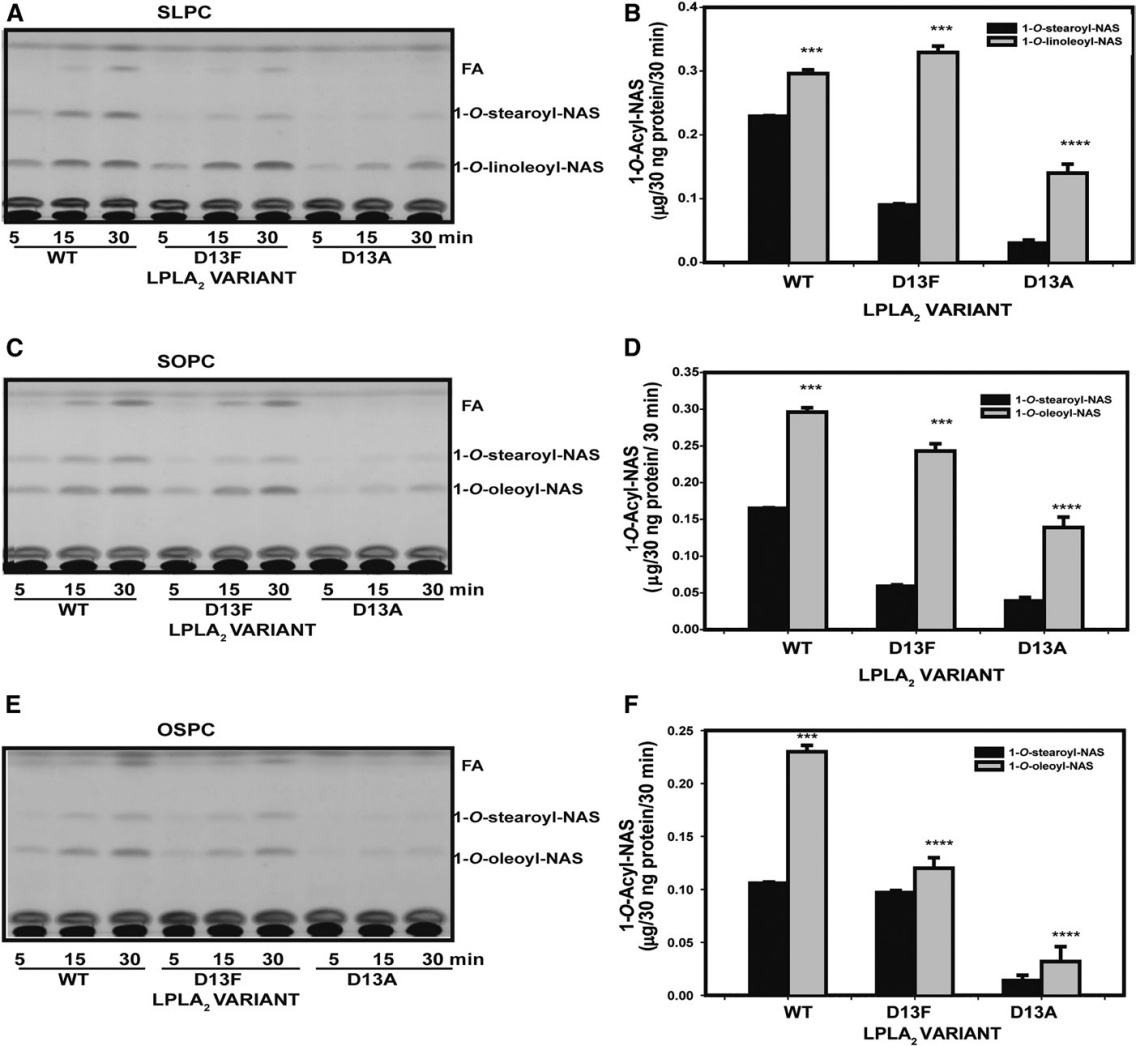
LPLA_2_ variant (WT, D13F, D13A) transacylase activities with SLPC, SOPC and OSPC at pH 4.5. Liposomes containing of SLPC, SOPC, or OSPC as substrates with sulfatide and NAS were incubated with purified LPLA_2_ variants for 5, 15, and 30 min at 37°C. The transacylase activities of different LPLA_2_ variants at 30 min using SLPC (A and B), SOPC (C and D), and OSPC (E and F) as substrate. The histogram values represent the mean ± SD (n = 3), *P < 0.05, **P < 0.01, ***P < 0.001.

Because the side chain of Asp13 contributes primarily to Track A in the active site, its structure and/or protonation state could contribute to the positional selectivity of the enzyme during acyl transfer. SLPC, SOPC, and OSPC in liposomes were thus first assayed at pH 4.5. SLPC and SOPC have the same saturated stearoyl *sn*-1 chain (C18:0), but vary in unsaturation on the *sn*-2 acyl chain (C18:2 vs. C18:1, respectively). OSPC has its acyl chains in opposite configuration from SOPC. When the LPLA_2_ variants were assayed with SLPC liposomes, both 1-*O*-stearoyl-NAS and 1-*O*-linoleoyl-NAS were formed (**Fig. 4A**, B), but the *sn*-2 linoleoyl group was favored as the scissile fatty acid no matter which variant was assayed, with greater selectivity exhibited by the D13F variant compared with WT or D13A. Using either SOPC or OSPC liposomes, 1-*O*-oleoyl-NAS was favored as the product over 1-*O*-stearoyl NAS. However, this selectivity was significantly diminished for the D13F variant (Fig. 4E, F). Although the acyltransferase activity of the D13A variant was significantly lower compared with the WT and D13F variant, a similar pattern of 1-*O*-acylceramide products was observed with this variant with respect to the unsaturated acyl groups being favored as scissile groups regardless of their *sn*-1 versus *sn*-2 position.

**Fig. 5.**
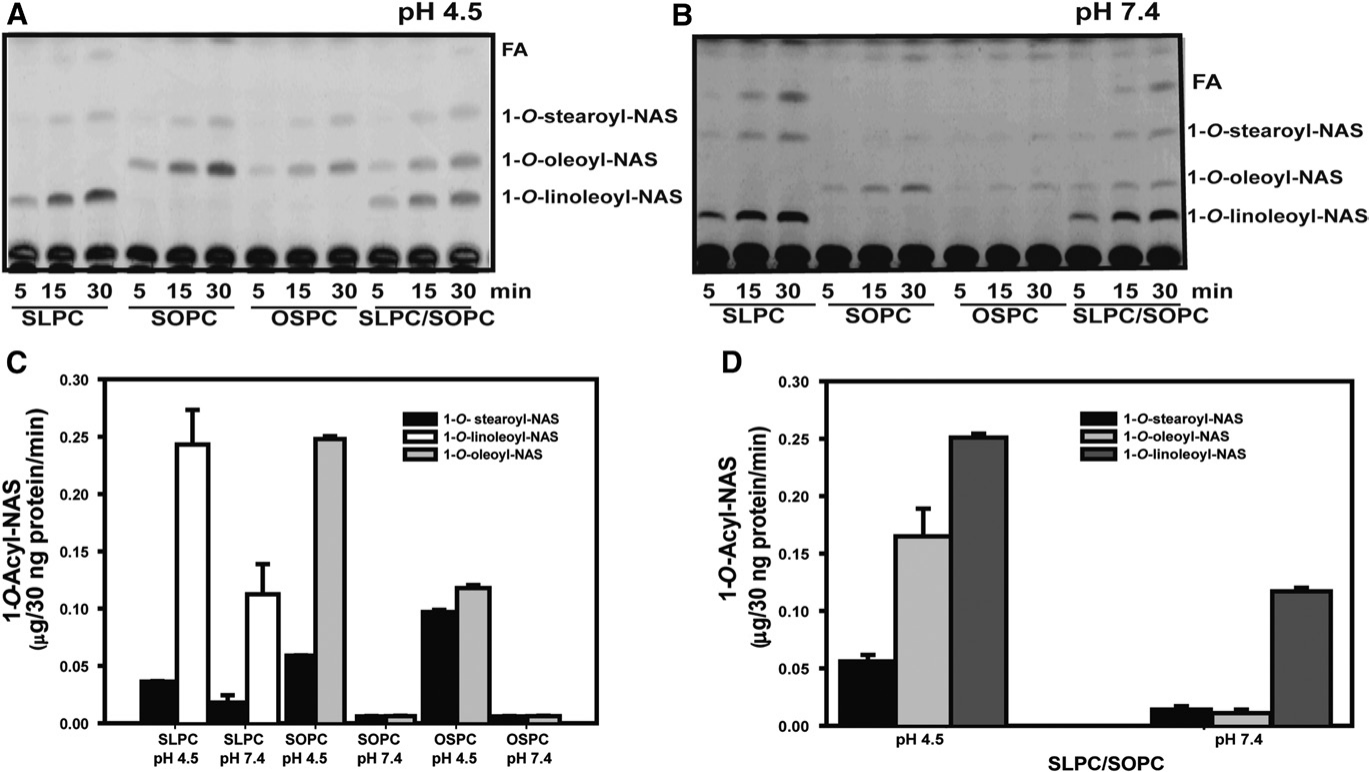
LPLA_2_-D13F transacylase activity with SLPC, SOPC, and OSPC substrates at pH 4.5 and 7.4. Liposomes containing SLPC, SOPC, OSPC, and SLPC/SOPC (1:1ratio) along with sulfatide, and NAS (10:1:3 molar ratio) were incubatedwith 30 ng protein of the D13F variant for 5, 15, and 30 min at 37°C at pH 4.5 and 7.4. The reaction 1-*O*-Acyl-NAS products were separated by HPTLC (A and B, respectively). The expression of *sn*-2/*sn*-1 transacylase activity of D13F at 30 min at pH 4.5 and pH 7.4 using SLPC, SOPC, OSPC (C), and mixed liposomes SLPC/SOPC (D) for acyl donor. The graph values represent the mean ± SD (n = 3) per time point using a *t*-test, *P < 0.05, **P < 0.01, ***P < 0.001.

The lipid substrate selectivity of the most active variant, D13F, was then compared at pH 4.5 and 7.4 using liposomes contained of phospholipids (SLPC, SOPC, OSPC)/sulfatide/NAS (10:1:3 molar ratio). To test LPLA_2_ substrate selectivity when more than two different fatty acids were present, a 1:1 mixture of SLPC and SOPC (SLPC/SOPC)/sulfatide/ NAS liposomes (10:1:3 molar ratio) was used. PLA_1_ and PLA_2_ activities were observed under all conditions (**Fig. 5**). Interestingly, at pH 7.4, D13F demonstrated a marked preference for *sn*-2 linoleoyl (C18:2) groups, with comparatively little 1-*O*-stearoyl- and 1-*O*-oleoyl-NAS formed (Fig. 5B).

### Positional selectivity of the D13F variant for fatty acyl chain length and unsaturation

**Fig. 6.**
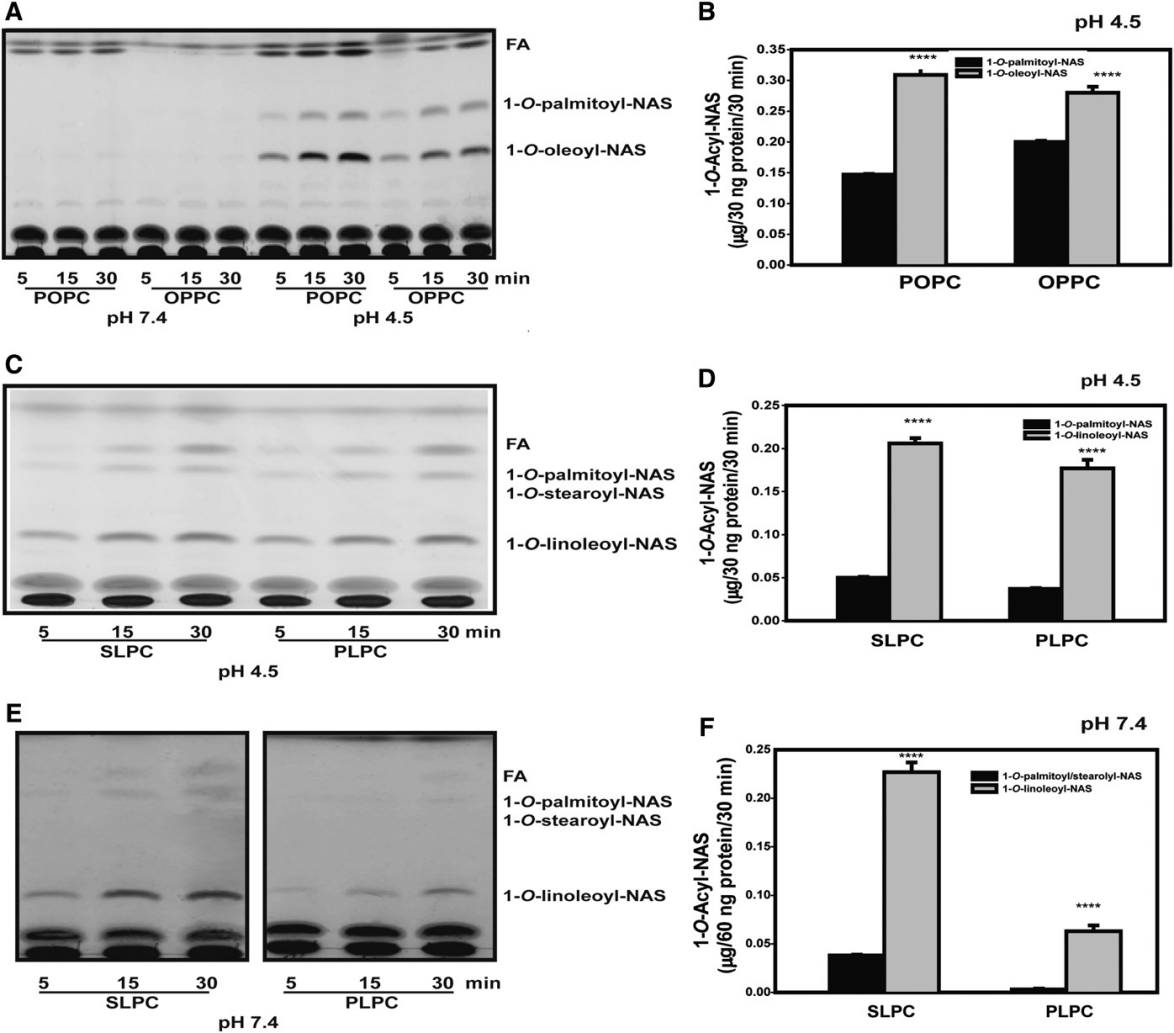
LPLA2 variant D13F transacylase activity with PLPC, POPC, and OPPC at pH 4.5 and 7.4. Liposomes containing of SLPC PLPC, POPC, and OPPC/sulfatide/ NAS (10:1:3 molar ratio) were incubated with purified D13F variant (30 ng, pH 4.5; 60 ng, pH 7.4) for 5, 15, and 30 min at 37°C. The reaction products were separated by argentation HPTLC using a solvent system consisting of chloroform/acetic acid/methanol (90:5:1). All histograms represent the mean of the LPLA_2_ transacylase activity ± SD (n = 3). A, B: 1-*O*-Acyl production of D13F using liposomes consisting of POPC and OPPC at pH 4.5 and 7.4. C, D: 1-*O*-Acyl production of D13F at pH 4.5 using liposomes consisting of SLPC or PLPC/sulfatide/NAS (10:1:3 molar ratio). E, F: Same conditions as in C and D except the transacylation reaction was conducted at pH 7.4. The graph values represent the mean ± SD (n = 3) per time point using a *t*-test, *P < 0.05, **P < 0.01, ***P < 0.001.

The influence of the D13F variant on acyl chain length was studied using phosphatidylcholine substrates containing palmitoyl (C16:0) groups in the *sn*-1 or *sn*-2 positions (POPC and OPPC), as well as phospholipids containing linoleic (C18:2) acid in the *sn*-2 position (PLPC, SLPC). When POPC or OPPC were used as substrates of the D13F variant, there was no observed activity at pH 7.4 but as expected the unsaturated oleoyl group was preferred at pH 4.5 (**Fig. 6A**, B).

The unsaturation of the *sn*-2 fatty acyl group in POPC was increased by using PLPC (C16:0, 18:2) as a substrate, which resulted in the formation of both 1-*O*-linoleoyl NAS and 1-*O*-palmitoyl NAS at both pH 4.5 and 7.4 (Fig. 6C–F), confirming a preference for hydrolysis and transacylation of the unsaturated *sn*-2 acyl group. When SLPC (C18:0, 18:2) and PLPC (C16:0, 18:2) were used as substrates at pH 4.5, the total formation of 1-*O*-acyl-NAS from SLPC was slightly higher than that of PLPC (Fig. 6C, D). When the same substrates were studied at pH 7.4, the formation of the 1-*O*-linoleoyl NAS produced from PLPC was four times less than that produced from SLPC (Fig. 6E, F), suggesting that the selectivity of the D13F variant also depends on the fatty acyl chain length.

When dimyristoyl phosphatidylcholine (C14:0, 14:0) was used as substrate, negligible activity was observed with WT and D13F variants at either pH (data not shown). When OMPC was used, the WT, D13F, and D13A variants showed results similar to OPPC at pH 4.5 with no activity at pH 7.4 (data not shown). These results also suggest that D13F LPLA_2_ variant displays preference for unsaturated acyl groups in *sn*-2 position of phosphatidylcholines for the transfer reaction, and that preference increased with length of the fatty acid occupying *sn*-1 position, as well as with the unsaturation of the fatty acid of *sn*-2 position. We also studied polyunsaturated long chain acyl groups at the *sn*-2 position of the phospholipid, including arachidonic (C20:4) and docosahexaenoic acid (C22:6). The positional specificity of LPLA_2_ for the *sn*-2 of phospholipids appears to be lessened by the polyunsaturated long chain acyl group at the *sn*-2 position, particularly when an arachidonoyl group is present. When PDPC having C22:6 acyl chain at the *sn*-2 position was used, LPLA_2_ acted on the sn-2 position preferentially but maintained sizable activity toward the sn-1 fatty acid. The D13F LPLA_2_ variant showed lower LPLA_2_ activity when mix liposomes containing linoleic acids were used (supplemental Fig. S1).

### Role of the phospholipid head group on substrate selectivity

We considered whether the glycerophospholipid head group contributed to the fatty acyl group selectivity via interactions with the Asp13 position. When liposomes containing SOPC, SOPE, SOPS, SOPG, or SOPA/sulfatide/NAS (10:1:3 molar ratio) were used at pH 4.5 as the acyl donor for either WT or D13F variants, both 1-*O*-stereoyl-NAS and 1-*O*-oleoyl-NAS were formed (supplemental Fig. S2). The best acyl donors were SOPS, and SOPG, likely due to the increased negative charge of their respective liposomes. Notably, the D13F variant exhibited more selectivity for 1-*O*-oleoyl-NAS (supplemental Fig. S2B, D), perhaps due to steric constraints imposed on Track A by its bulkier side chain. When the reaction was performed at pH 7.4, only SOPG and SOPA exhibited measurable activity with WT (supplemental Fig. S3A, B), but the D13F variant regained activity against all tested phospholipids, suggesting that this variant exhibits stronger interactions with its substrates in general, perhaps through increased acyl chain interactions but also possibly because there is no charge repulsion between Asp13 and the phosphate in the head group. When POPC, POPE, POPG, POPS and POPA/sulfatide/NAS (10:1:3 molar ratio) were used as acyl donors at pH 4.5, POPS, POPC, and POPG were the preferred substrates. Thus, the selectivity of LPLA_2_ for *sn*-1, and *sn*-2 position does not depend on a head group. However, the rate of hydrolysis is dependent on the phospholipid head group.

### Acylation of HAG, HG, and other biosynthetic lipophilic alcohols by the D13F variant

**Fig. 7.**
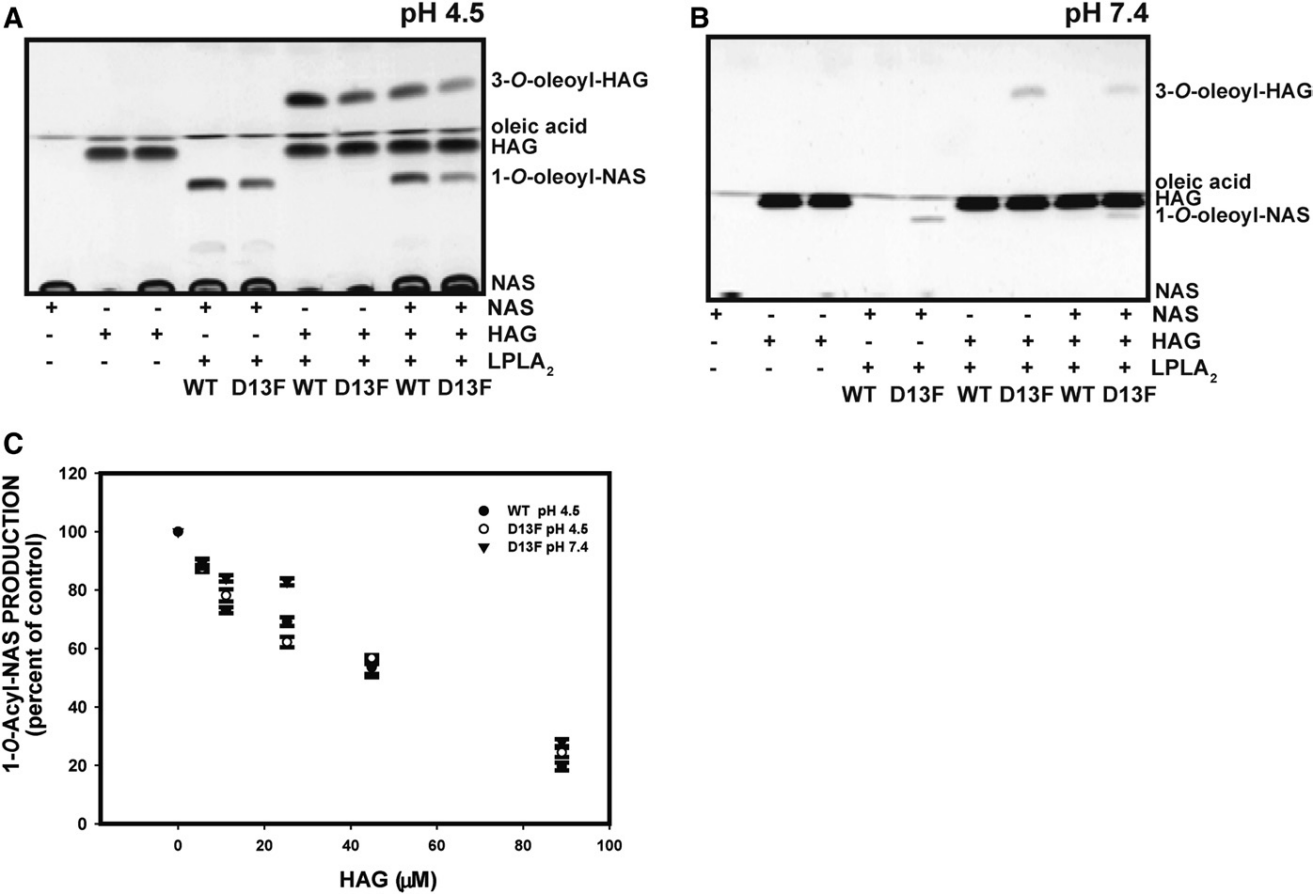
Acylation of lipophilic alcohols by LPLA_2_. A: The formation of 3-*O*-acyl-HAG (1-*O*-hexadecyl-2-acetyl-3-oleoyl-sn-glycerol) and 1-*O*-acyl-NAS by WT and D13F LPLA_2_ at pH 4.5 were compared. The reaction mixtures consisted of 48 mM Na-citrate (pH 4.5), LPLA2 (30 ng), and liposomes consisting of DOPC, sulfatide, and acceptor (NAS, HAG, or NAS and HAG) with a molar ratio of 10:1:3.25 and in a total volume of 500 µl After the incubation, the lipids were extracted, applied to an HPTLC plate, and developed in a solvent system consisting of chloroform/acetic acid 96/4, v/v. B: The formation of 3-*O-*acyl- HAG and 1-*O*-acyl-NAS by WT and D13F LPLA_2_ at pH 4.5 and 7.4 were compared. The reaction mixture was the same as in A except for the use of HEPES buffer (50 mM) and 60 ng of enzyme. C: The concentration dependent inhibition of 1-*O*-acyl-NAS by HAG. The graph values represent the mean ± SD (n = 3) per time point using a *t*-test, *P < 0.05, **P < 0.01, ***P < 0.001.

NAS is not the only acceptor for LPLA_2_ transacylation. Other acceptors include ethanol derivatives with one long aliphatic chain and one short neutral residue, such as methyl or acyl group in the C2 position (28). A number of natural and synthetic lipophilic alcohols that have similar structure to NAS were tested to determine whether they could serve as the acceptor in the transacylase reaction of LPLA_2_ and whether the D13F variant has impact on selectivity. HAG is an alkylacylglycerol with a primary alcohol group at the C3 position and is structurally very similar to NAS. When liposomes consisting of DOPC/sulfatide/HAG were used, two products were formed, oleic acid and 1-*O*-hexadecyl-2-3-oleoyl-*sn*-glycerol at either pH 4.5 or 7.4 (**Fig. 7A**, B). When HAG and NAS were simultaneously present in liposomes, HAG inhibited the acylation of NAS in a concentration dependent manner (Fig. 7C).

Other primary alcohols, including 1-*O*-hexadecyl-rac-glycerol, oleoylethanolamide, and andamide were also assayed. Similar to HAG, we did not observe major differences in the products or their ratios between WT and the D13F variant at pH 4.5 (data not shown). As with NAS as an acceptor, the D13F variant was also able to acylate all lipophilic alcohols at pH 7.4. Therefore, the D13F variant showed no effect on the acceptor selectivity and this position is seeming unlikely to contribute to the acceptor binding.

### Molecular mechanisms for LPLA_2_ substrate selectivity

**Fig. 8.**
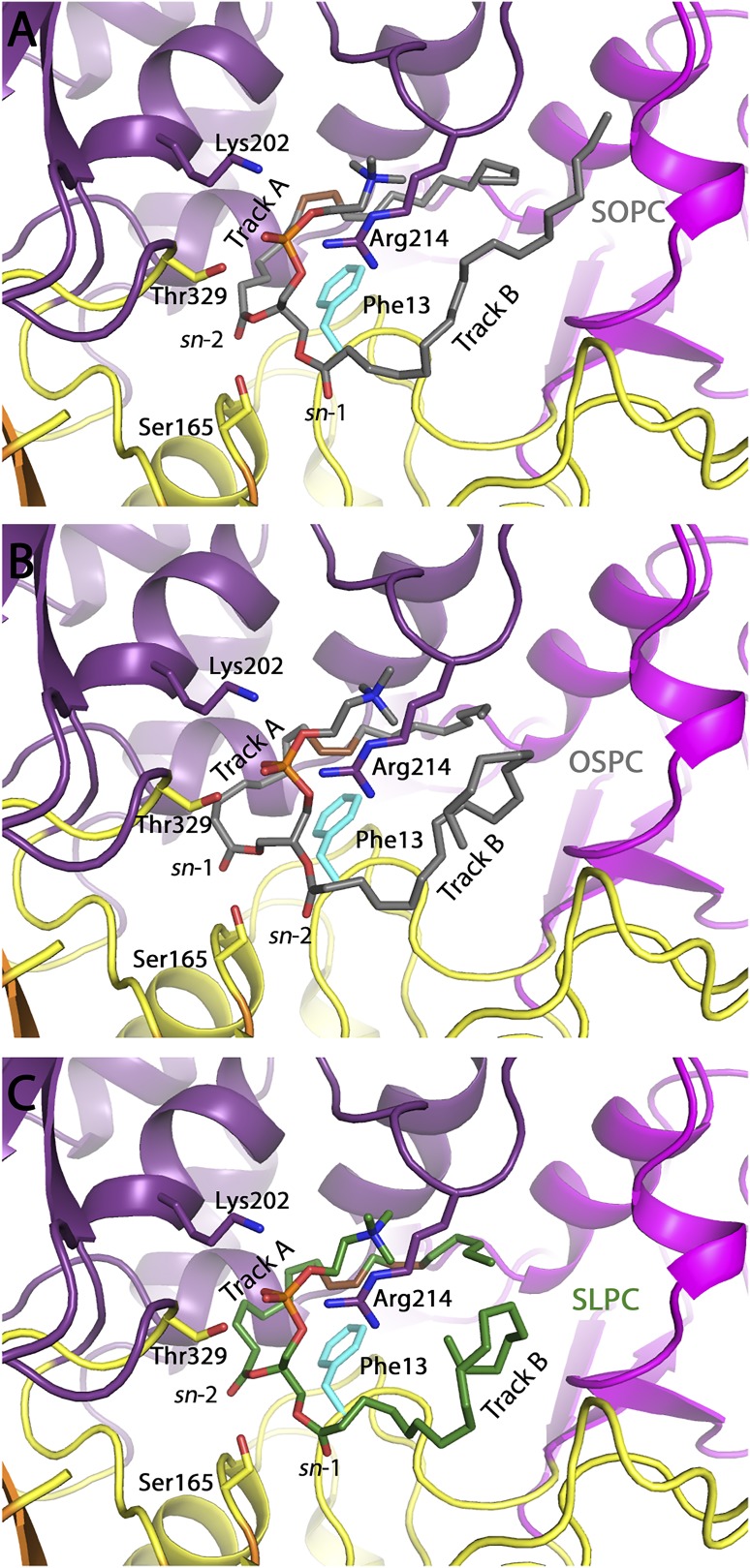
A: Overall fold of LPLA_2_ showing the α/β hydrolase (yellow with orange β-sheets), membrane binding (magenta), and cap (purple) domains. SOPC (gray carbons) is shown with track A occupied by the *sn*-2 chain and the *cis* double bond in black. B: Docking of SOPC and OSPC into a model of LPLA2 D13F. OSPC (gray carbons) is shown with track A occupied by the *sn*-1 chain. The *cis* double bond of the lipid tail is shown in brown. The sidechain of Phe13 is shown in cyan. C: Docking of SLPC into a model of LPLA2 D13F. SLPC (green carbons) is shown with track A occupiesd by the *sn*-2 chain. The *cis* double bonds of the lipid tail are shown in brown. The side chain of Phe13 is shown in cyan.

Our previously reported data (13) and the data presented herein shows that LPLA_2_ can cleave both *sn*-1 and *sn*-2 fatty acyl groups on a variety of glycerophospholipids, and largely does so independent of the head group and the acyl acceptor, with preference typically given to the more unsaturated acyl chain, provided there are not four or more unsaturated positions (supplemental Fig. S1). Substitution of Asp13 seems to have little impact on this selectivity other than to impart additional bias against saturated acyl chains in the *sn*-1 position (e.g., Fig. 3 and supplemental Fig. S2). Based on these data, we modeled phospholipids with either *sn*-1 or *sn*-2 fatty acyl groups in track A for each of the phospholipids that are substrates for the lipase. SOPC and OSPC were docked into D13F variant in order to demonstrate that it is possible for the unsaturated acyl chain (oleoyl) to serve as the donor for transacylation regardless of its *sn*-1 or *sn*-2 attachment (**Fig. 8A**, B). During transacylation, the chain located in track A forms an acyl intermediate with Ser165. Indeed, docking showed that when the unsaturated chain is oriented in track A. The *cis* double bond creates a kink in the lipid tail to curve around the side chain of Asp13. Having a phenylalanine in place of an aspartate would allow for additional π-π interactions between the aromatic ring and double-bond, potentially explaining why the D13F variant preferentially transfers the oleoyl acyl chain regardless of whether it is in the *sn*-1 or *sn*-2 position. Docking with SLPC (Fig. 8C) suggests that the additional double bond of the linoleoyl chain will also be able to form additional van der Waals interactions with Phe13.

**Fig. 9.**
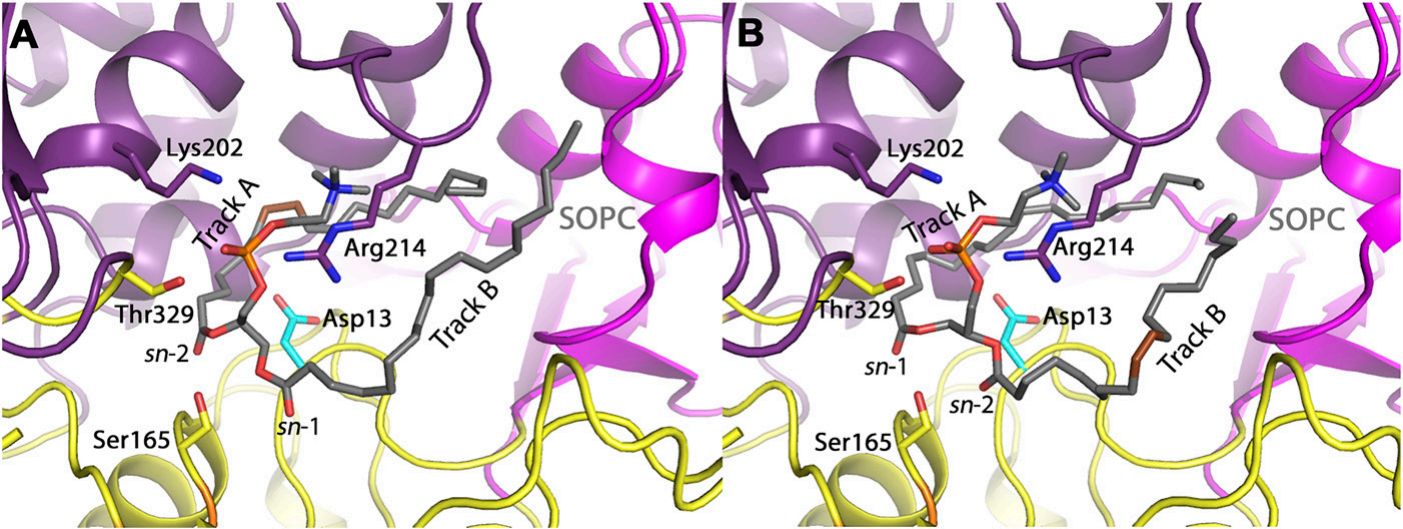
Docking of SOPC into LPLA2 WT. A: SOPC (gray carbons) is shown with track A occupied by the *sn*-2 chain. B: SOPC (gray carbons) is shown with track A occupied by the *sn*-1 chain. The *cis* double bond of the lipid tail is shown in brown. The sidechain of Asp13 is shown in cyan.

Docking of SOPC into LPLA_2_ WT was performed to model how the *sn*-1 chain or *sn*-2 chain (**Fig. 9**) could be oriented to occupy track A and interact with Asp13. WT only shows a slight preference for the *sn*-2 position, perhaps because Asp13 makes more limited interactions with the acyl chain in track A. Docking of lipid substrates with various head groups was also performed (supplemental Fig. S4). In each case, the phosphate groups of the head group aligned very closely and formed interactions with conserved residues Lys202 and Arg212 (and in most cases Thr329). Beyond that the remainder of the head group formed minimal interactions with the protein. Therefore, we expect that the interactions with the head group and enzyme should not influence the substrate specificity, consistent with our results. Rather, other physical properties of liposomes that are controlled by the head group are likely to play a dominant role. This might include electrostatic interactions between anionic phospholipids and cationic residues on the enzyme (9) and alterations in the phase properties of the bilayer.

## DISCUSSION

Glycerophospholipids are highly diverse structures due not only to differences in their head groups but also to asymmetry in their acyl chains. The esterification of saturated and monounsaturated fatty acids typically occurs at the *sn*-1-position whereas unsaturated fatty acids are generally restricted to the *sn*-2-position. Although the composition of phospholipids may be determined in part by de novo synthetic pathways, remodeling of membranes by phospholipases and acyl-CoA:lysophospholipases is also important in maintaining this asymmetry (29, 30). The basis by which enzymes can distinguish between saturated and unsaturated acyl chains has thus been the subject of considerable attention (31).

Phospholipases are defined as A_1_ or A_2_ based on their ability to catalyze the hydrolysis of *sn*-1 versus *sn*-2 fatty acyl groups. Many PLA_2_ enzymes display strict specificity toward the *sn*-2 fatty acyl group based on the lyso-lipid formed. However, because acyl groups may migrate between hydroxyl groups on lyso-lipids, strict proof of the fatty acyl specificity of many PLA_2_ family members is often lacking. Because LPLA_2_ is both a phospholipase and transacylase, the determination of which acyl group represents the scissile fatty acid can be unambiguously determined due to the ability to distinguish between the 1-*O*-acyl-ceramides formed.

Our current studies of an unusual charged amino acid found in the active site of LPLA_2_ demonstrate that this residue, Asp13, has the ability to tune the activity of the enzyme via its protonation state. When this fatty acid is substituted with a phenylalanine, the pH dependence is less pronounced and both phospholipase and transacylase activities can be observed at pH 7.4 with DOPC as the substrate. Because the activity of WT and D13F LPLA_2_ are essentially the same at pH 4.5, the boost in activity observed at higher pH with D13F is unlikely due to additional interactions formed by the phenylalanine side chain with phospholipid substrates.

Our biochemical and modeling results also show that the residue at position 13, whose side chain contributes to track A, is also able to tune acyl chain preference, likely through direct interactions with the acyl chain, consistent with our prediction that the scissile fatty acyl group always resides in track A of the enzyme. When the unsaturated acyl chain is in the *sn*-2 position, D13F variant enhances selectivity for transfer of the unsaturated chain. Conversely, D13F is less selective than WT for unsaturated acyl chains when they are in the *sn*-1 position (Figs. 4, 5). Although modeling indicates that phospholipids can dock with either acyl chain in track A, the molecular basis for the opposing effects of D13F on selectivity for unsaturated acyl chains is unclear.

Our data shows that the identity of residue 13 does not seem to influence selectivity for acyl acceptors or the phospholipid head group. The former result is somewhat surprising given its proximity to the acyl transfer site. The head group of the glycerophospholipid, on the other hand, docks relatively far away from Asp13. Electrostatic repulsion between the phosphate group common to all phospholipids and deprotonated Asp13 may, however, also contribute to its lower activity at high pH.

The closely related enzyme LCAT has a cysteine instead of aspartate in the analogous position (Cys31). This is the most prominent difference between LCAT and LPLA_2_ in the active site other than the dynamic lid that partially covers the hydrophobic bowl of the active site. However, neither the D13C substitution nor the D13C substitution along with other conservative changes in the active site (ToLCC) were as adept as D13F in recovering acyltransferase activity at neutral pH where LCAT is the most active (although D13C did have low measurable activity). D13C and ToLCC were also unable to demonstrate activity against a cholesterol-like substrate in a synthetic HDL-based activity assay. These results, along with a lack of obvious effects on acyl acceptor identity with the D13F variant (Fig. 7), indicate that acyl acceptor preference may be most strongly dictated by the dynamic lid element in the LCAT/LPLA_2_ family. Conversely, their selectivity for glycerophospholipid substrates is strongly dictated by interactions by features within the active site cavity, including track A and the identity of the residue at position 31/13, respectively, with the acyl chains.

## Supplementary Material

Supplemental Data

## References

[1] Shayman J. A., Abe A., and Radin N. S. 1996 A new pathway for ceramide metabolism: the catalytic esterification of ceramide to form 1-O-acylceramide by a novel phospholipase A2. J. Am. Soc. Nephrol. 7: A2196–A2196.

[2] Abe A., and Shayman J. A. 1998 Purification and characterization of 1-O-acylceramide synthase, a novel phospholipase A(2) with transacylase activity. J. Biol. Chem. 273: 8467–8474. 9525960 10.1074/jbc.273.14.8467

[3] Hiraoka M., Abe A., and Shayman J. A. 2002 Cloning and characterization of a lysosomal phospholipase A(2), 1-O-acylceramide synthase. J. Biol. Chem. 277: 10090–10099. 11790796 10.1074/jbc.M111977200

[4] Shayman J. A., Kelly R., Kollmeyer J., He Y., and Abe A. 2011 Group XV phospholipase A(2), a lysosomal phospholipase A(2). Prog. Lipid Res. 50: 1–13. 21074554 10.1016/j.plipres.2010.10.006 PMC3039127

[5] Hiraoka M., Abe A., Lu Y., Yang K., Han X., Gross R. W., and Shayman J. A. 2006 Lysosomal phospholipase A2 and phospholipidosis. Mol. Cell. Biol. 26: 6139–6148. 16880524 10.1128/MCB.00627-06 PMC1592808

[6] Paduraru C., Bezbradica J. S., Kunte A., Kelly R., Shayman J. A., Veerapen N., Cox L. R., Besra G. S., and Cresswell P. 2013 Role for lysosomal phospholipase A2 in iNKT cell-mediated CD1d recognition. Proc. Natl. Acad. Sci. USA. 110: 5097–5102. 23493550 10.1073/pnas.1302923110 PMC3612615

[7] Schneider B. E., Behrends J., Hagens K., Harmel N., Shayman J. A., and Schaible U. E. 2014 Lysosomal phospholipase A2: a novel player in host immunity to Mycobacterium tuberculosis. Eur. J. Immunol. 44: 2394–2404. 24825529 10.1002/eji.201344383

[8] Abe A., and Shayman J. A. 2009 The role of negatively charged lipids in lysosomal phospholipase A2 function. J. Lipid Res. 50: 2027–2035. 19321879 10.1194/jlr.M900008-JLR200 PMC2739751

[9] Glukhova A., Hinkovska-Galcheva V., Kelly R., Abe A., Shayman J. A., and Tesmer J. J. 2015 Structure and function of lysosomal phospholipase A2 and lecithin:cholesterol acyltransferase. Nat. Commun. 6: 6250. 25727495 10.1038/ncomms7250 PMC4397983

[10] Abe A., Hiraoka M., and Shayman J. A. 2007 A role for lysosomal phospholipase A2 in drug induced phospholipidosis. Drug Metab. Lett. 1: 49–53. 19356018 10.2174/187231207779814292

[11] Abe A., Hiraoka M., Ohguro H., Tesmer J. J., and Shayman J. A. 2017 Preferential hydrolysis of truncated oxidized glycerophospholipids by lysosomal phospholipase A2. J. Lipid Res. 58: 339–349. 27993948 10.1194/jlr.M070730 PMC5282950

[12] Abe A., Kelly R., Kollmeyer J., Hiraoka M., Lu Y., and Shayman J. A. 2008 The secretion and uptake of lysosomal phospholipase A2 by alveolar macrophages. J. Immunol. 181: 7873–7881. 19017977 10.4049/jimmunol.181.11.7873

[13] Abe A., Hiraoka M., and Shayman J. A. 2006 Positional specificity of lysosomal phospholipase A(2). *J. Lipid Res.* **47:** 2268–2279. 10.1194/jlr.M600183-JLR200 16837646

[14] Manthei K. A., Ahn J., Glukhova A., Yuan W., Larkin C., Manett T. D., Chang L., Shayman J. A., Axley M. J., Schwendeman A., 2017 A retractable lid in lecithin:cholesterol acyltransferase provides a structural mechanism for activation by apolipoprotein A-I. J. Biol. Chem. 292: 20313–20327. 29030428 10.1074/jbc.M117.802736 PMC5724016

[15] Kuivenhoven J. A., Pritchard H., Hill J., Frohlich J., Assmann G., and Kastelein J. 1997 The molecular pathology of lecithin:cholesterol acyltransferase (LCAT) deficiency syndromes. J. Lipid Res. 38: 191–205. 9162740

[16] Ollis D. L., Cheah E., Cygler M., Dijkstra B., Frolow F., Franken S. M., Harel M., Remington S. J., Silman I., Schrag J., 1992 The alpha/beta hydrolase fold. Protein Eng. 5: 197–211. 1409539 10.1093/protein/5.3.197

[17] Jauhiainen M., Stevenson K. J., and Dolphin P. J. 1988 Human plasma lecithin-cholesterol acyltransferase. The vicinal nature of cysteine 31 and cysteine 184 in the catalytic site. J. Biol. Chem. 263: 6525–6533. 3129428

[18] Francone O. L., and Fielding C. J. 1991 Effects of site-directed mutagenesis at residues cysteine-31 and cysteine-184 on lecithin-cholesterol acyltransferase activity. Proc. Natl. Acad. Sci. USA. 88: 1716–1720. 1848009 10.1073/pnas.88.5.1716 PMC51095

[19] Abe A., Shayman J. A., and Radin N. S. 1996 A novel enzyme that catalyzes the esterification of N-acetylsphingosine. Metabolism of C-2-ceramides. J. Biol. Chem. 271: 14383–14389. 8662981 10.1074/jbc.271.24.14383

[20] Homan R., Esmaeil N., Mendelsohn L., and Kato G. J. 2013 A fluorescence method to detect and quantitate sterol esterification by lecithin:cholesterol acyltransferase. Anal. Biochem. 441: 80–86. 23851343 10.1016/j.ab.2013.06.018 PMC3822574

[21] Li D., Gordon S., and A. T. Remaley 2015 Apolipoprotein mimetic peptides for stimulating cholesterol efflux. *In* Apolipoprotein mimetics in management of human disease. G. M. Anantharamaiah and D. Goldberg, editors. Springer International Publishing, New York. 29–42.

[22] Piper D. E., Romanow W. G., Gunawardane R. N., Fordstrom P., Masterman S., Pan O., Thibault S. T., Zhang R., Meininger D., Schwarz M., 2015 The high-resolution crystal structure of human LCAT. J. Lipid Res. 56: 1711–1719. 26195816 10.1194/jlr.M059873 PMC4548775

[23] Adams P. D., Afonine P. V., Bunkoczi G., Chen V. B., Davis I. W., Echols N., Headd J. J., Hung L. W., Kapral G. J., Grosse-Kunstleve R. W., 2010 PHENIX: a comprehensive Python-based system for macromolecular structure solution. Acta Crystallogr. D Biol. Crystallogr. 66: 213–221. 20124702 10.1107/S0907444909052925 PMC2815670

[24] Trott O., and Olson A. J. 2010 AutoDock Vina: improving the speed and accuracy of docking with a new scoring function, efficient optimization, and multithreading. J. Comput. Chem. 31: 455–461. 19499576 10.1002/jcc.21334 PMC3041641

[25] Semisotnov G. V., Rodionova N. A., Razgulyaev O. I., Uversky V. N., Gripas A. F., and Gilmanshin R. I. 1991 Study of the “molten globule” intermediate state in protein folding by a hydrophobic fluorescent probe. Biopolymers. 31: 119–128. 2025683 10.1002/bip.360310111

[26] Bonelli F. S., and Jonas A. 1989 Reaction of lecithin cholesterol acyltransferase with water-soluble substrates. J. Biol. Chem. 264: 14723–14728. 2768238

[27] Hiraoka M., Abe A., and Shayman J. A. 2005 Structure and function of lysosomal phospholipase A2: identification of the catalytic triad and the role of cysteine residues. J. Lipid Res. 46: 2441–2447. 16106046 10.1194/jlr.M500248-JLR200

[28] Abe A., Hiraoka M., and Shayman J. A. 2007 The acylation of lipophilic alcohols by lysosomal phospholipase A2. J. Lipid Res. 48: 2255–2263. 17626977 10.1194/jlr.M700277-JLR200

[29] Schaloske R. H., and Dennis E. A. 2006 The phospholipase A2 superfamily and its group numbering system. Biochim. Biophys. Acta. 1761: 1246–1259. 16973413 10.1016/j.bbalip.2006.07.011

[30] Shindou H., and Shimizu T. 2009 Acyl-CoA:lysophospholipid acyltransferases. J. Biol. Chem. 284: 1–5. 18718904 10.1074/jbc.R800046200

[31] Lands W. E. 2000 Stories about acyl chains. Biochim. Biophys. Acta. 1483: 1–14. 10601692 10.1016/s1388-1981(99)00177-8

[32] Vivoli M., Novak H. R., Littlechild J. A., and Harmer N. J. 2014 Determination of protein-ligand interactions using differential scanning fluorimetry. J. Vis. Exp. 51809. 25285605 10.3791/51809 PMC4692391

