# Supplementary material for: Determinants of pH profile and acyl chain selectivity in lysosomal phospholipase A2
Source: J Lipid Res. 2018 May 3;59(7):1205–18. doi: 10.1194/jlr.M084012 (PMC6027918; doi:10.1194/jlr.M084012)
Supplement: Supplemental Data [file supp_59_7_1205__index.html]

Determinants of pH profile and acyl chain selectivity in lysosomal phospholipase A2 — Determinants of pH profile and acyl chain selectivity in lysosomal phospholipase A2 — Supplemental Data 

# Determinants of pH profile and acyl chain selectivity in lysosomal phospholipase A2

## Supplemental Data

- Supplemental figure 1 (.pdf, 31 KB) - Positional selectivity of D13F variant with SAPC, PAPC, PDPC and mixed liposomes with SAPC/SOPC or SLPC/SOPC/SAPC at pH 4.5 and pH 7.4.
- Supplemental figure 3 (.pdf, 39 KB) - Transacylase activities of WT and D13F LPLA2 using phospholipids with different head groups at pH 7.4.
- Supplemental figure 4 (.pdf, 109 KB) - Comparison of lipid head group docking positions.
