# Supplementary material for: Determinants of pH profile and acyl chain selectivity in lysosomal phospholipase A2
Source: J Lipid Res. 2018 May 3;59(7):1205–18. doi: 10.1194/jlr.M084012 (PMC6027918; doi:10.1194/jlr.M084012)
Supplement: Supplemental Data [file 10.1194_M084012_jlr.M084012-1.pdf]

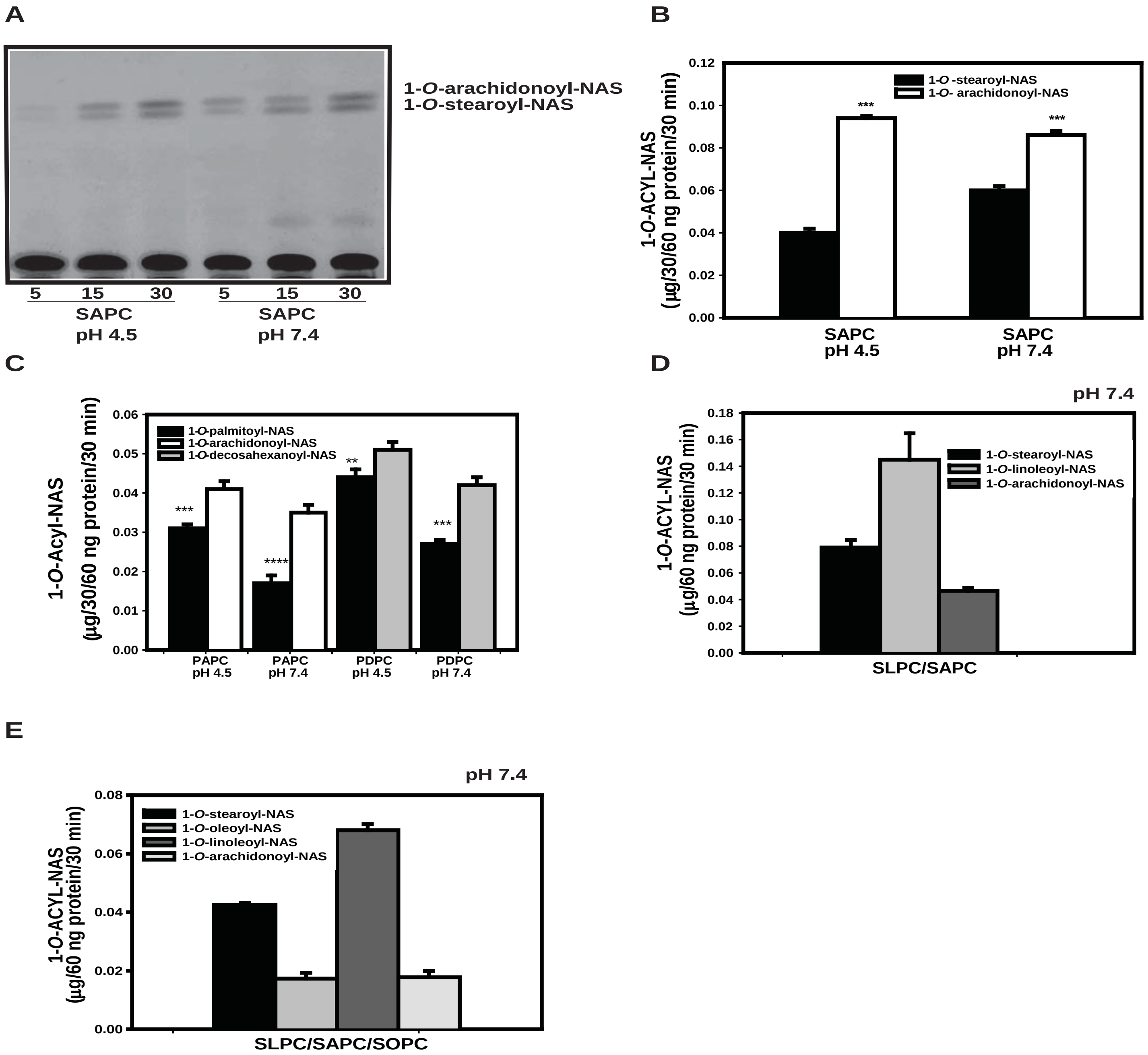

Supplemental Fig.1. Positional selectivity of D13F variant with SAPC, PAPC, PDPC and mixed liposomes with SAPC/SOPC or SLPC/SOPC/SAPC at pH 4.5 and pH 7.4. Liposomes consisting of the SAPC, PAPC, PDPC, or SAPC/SOPC or SLPC/SOPC/SAPC /sulfatide and NAS (10:1:3 molar ratio) were incubated with 30 ng (pH 4.5), or 60 ng (pH 7.4) purified protein for 5, 15, and 30 min at 37 ˚C. The reaction products were separated on HPTLC, and quantified by scanning the plate and the initial velocity was estimated. For separations of reaction products, two solvent systems were used. For separating arachidonoyl **(A, B, C)** or docosohexanoyl **(C)** products the solvent system consisted of chloroform/acetic acid (9:1 v/v). For separating the products of SAPC/SOPC or SLPC/SOPC/SAPC mix liposomes assays, the system consisted of chloroform/acetic acid/methanol (90:5:1v/v) and an argentation HPTLC **(D, E)**. All histograms represent mean ± SD (n=3) per point *p<0.05, **p<0.01, ***p<0.001 using *t* test
