# Supplementary material for: Determinants of pH profile and acyl chain selectivity in lysosomal phospholipase A2
Source: J Lipid Res. 2018 May 3;59(7):1205–18. doi: 10.1194/jlr.M084012 (PMC6027918; doi:10.1194/jlr.M084012)
Supplement: Supplemental Data [file 10.1194_M084012_jlr.M084012-2.pdf]

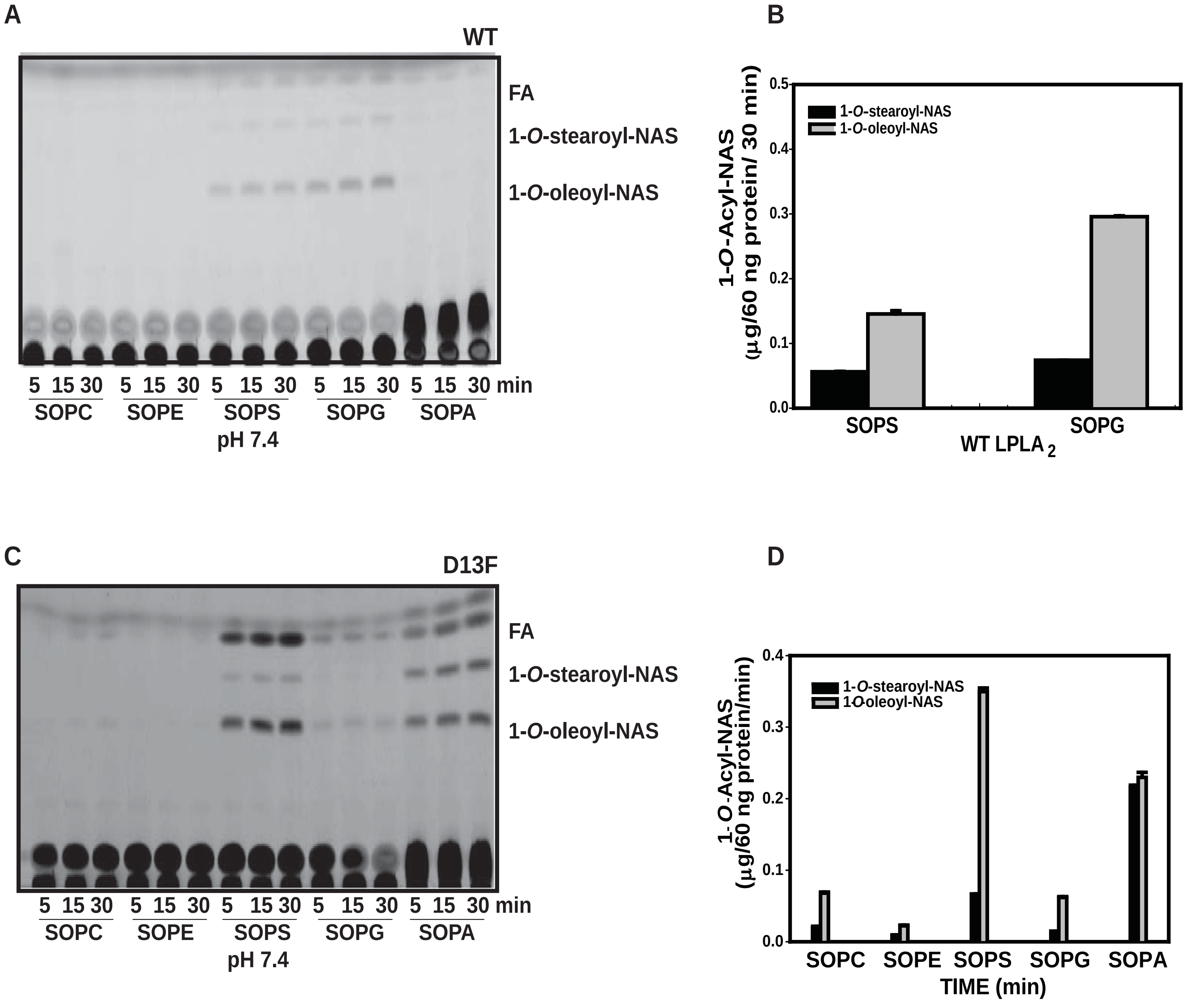

Supplemental Fig. 3. Transacylase activities of WT and D13F LPLA$_2$ using phospholipids with different head groups at pH 7.4. SOPC, SOPE, SOPS, SOPG, and SOPA/sulfatide/NAS containing liposomes mixed in a 10:1:3 molar ratio with sulfatide and NAS were incubated with 60 ng purified protein of WT (A, B) and D13F (C, D) variants in 50 mM HEPES pH 7.4 for 5, 15, 30 min at 37 ˚C. The reaction products were extracted and separated by argentation HPTLC using a solvent system chloroform/acetic acid/methanol (90:5:1). The reaction products 1-*O*-stearoyl NAS and 1-*O*-oleoyl NAS were quantified by scanning the plate, and the initial velocity was estimated. All histograms represent means ± SD (n=3).
