# Supplementary material for: Determinants of pH profile and acyl chain selectivity in lysosomal phospholipase A2
Source: J Lipid Res. 2018 May 3;59(7):1205–18. doi: 10.1194/jlr.M084012 (PMC6027918; doi:10.1194/jlr.M084012)
Supplement: Supplemental Data [file 10.1194_M084012_jlr.M084012-3.pdf]

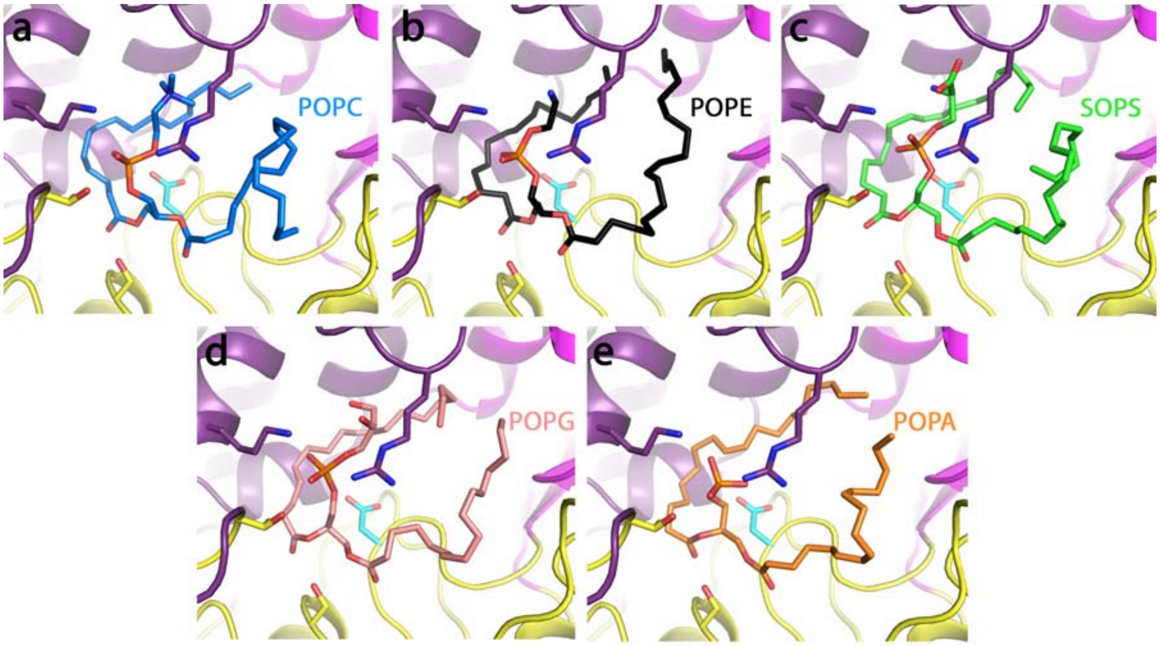

Figure S4. **Comparison of lipid head group docking positions.** (A) POPC (blue carbons), (B) POPE (black carbons), (C) SOPS (green carbons), (D) POPG (salmon carbons), and (E) POPA (orange carbons) were docked into $LPLA_2$ WT to compare the relative positions of the lipid head groups. The phosphate groups aligned very closely in all cases and were within hydrogen bond distance of Lys202 and Arg214. The side chains of the key residues are shown in purple sticks (Lys202, Arg214), yellow sticks (Thr329, Ser165), and cyan (Asp13).
